# The relationship between autism spectrum disorder and sleep

**DOI:** 10.5935/1984-0063.20210050

**Published:** 2021

**Authors:** Sandra Doria Xavier

**Affiliations:** Departamento de Psicobiologia, Universidade Federal de São Paulo (UNIFESP), São Paulo, Brazil.

## INTRODUCTION

This issue of *Sleep Science* contains 2 reports related to autism spectrum disorder (ASD), an extremely important and relatively frequent neurodevelopmental disorder. This condition is characterized by impaired reciprocal social interaction and communication, as well as restricted and repetitive patterns of behaviors or interest. It affects 1 in every 40 to 59 children in the United States, with prevalence rates doubling between 2000-2002 and 2010-2012^1-3^. Sleep disturbances are some of the marked challenges faced by young people with ASD, their families and caregivers. Approximately 50% to 80% of children and adolescents with ASD suffer from sleep problems^4^ in comparison to 20-30% in neurotypical (NT) children^4-7^.

The usual sleep-related complaints and symptoms among ASD children are insomnia, bedtime settling issues, sleep anxiety, night waking, poor sleep quality and sleep-disordered breathing^5,8^. Problems initiating and maintaining sleep are one the most common concurrent clinical complaints and are less likely to diminish with age compared with NT children^9,10^.

These sleep difficulties commonly faced by children with ASD can produce a significant decrease in the quality of life of all family members as a consequence of sleep deprivation. As sleep is a behavioral and emotional regulator, sleep fragmentation or deprivation can worsen behavioral disturbances in children with ASD^11-13^, possibly triggering disruptive or inflexible behavior and anxiety^14-20^. Greater variation in sleep duration and timing have been found to predict subsequent disruptive daytime behavior^21^. Parents of children with ASD and sleep problems may, thus, have to deal with 2 consequences: firstly, their child´s behavior problems derived from their sleep issues and secondly, the consequences of their own sleep deprivation.

One important question is whether the sleep difficulties of children with ASD also affect their siblings as well as their parents. Understanding the experiences of this population is essential as the relationship that an individual with ASD has with a sibling is typically their longest relationship, and it can have a substantial impact on emotional, behavioral, and psychological outcomes^22^. However, few studies have examined the sleep of the siblings of children with ASD (ASD-Sib). In this issue of Sleep Science, Naeen et al. provided very interesting data on the sleep of the immediate family of individuals with ASD. The authors investigated 64 children with ASD, 80 of their siblings and 80 NT children using a sleep-wake diary, a school sleep habit questionnaire and a childhood autism spectrum test. Surprisingly, the comparison revealed no significant differences between the children with ASD and their siblings, nor between these 2 groups and the control subjects in terms of their sleep profiles.

These results are contrary to those of some previous studies, demonstrating that this is a very complex issue, which probably depends on how siblings deal with the altered family functioning secondary to ASD^23^. In 2012, Chou et al. reported that ASD-Sib have a higher risk of early insomnia and parasomnias compared to NT children^24^. Shivers et al. (2019) published a meta-analysis about the degree to which ASD-Sibs function similarly or differently compared to siblings of NT people and to the siblings of individuals with other disabilities. They described that there were specific areas of functioning in which ASD-Sibs fared worse, such as internalizing behavior problems, psychological functioning, beliefs, social functioning, and the sibling relationship^25^. Recent data from Taylor et al.^26^ presented a genetic role for sleep issues in ASD siblings. They investigated etiological links between ASD and difficulties initiating and maintaining sleep in 15,279 child and adolescent ASD twin pairs. The authors found that monozygotic co-twins of ASD individuals were most at risk of difficulties initiating and maintaining sleep compared to the reference group, followed by dizygotic co-twins and full siblings. Their results suggest that shared genetic mechanisms could underlie ASD and sleep difficulties. The take home message of the study is that more attention needs to be paid to the care of the siblings of young individuals with ASD. They may not only be directly affected by the family concerns involving their siblings but also indirectly by a shared underlying genetic link.

In this issue of Sleep Science, Cebreros-Paniagua et al. investigated sleep microstructure in children with Asperger´s Syndrome (AS). According to the Diagnostic and Statistical Manual of Mental Disorders 5 (DSM-5)^27^, AS is part of the broad spectrum of neurodevelopmental disorders that comprise ASD. Different levels of severity ranging from childhood autism with language impairment to so-called “high level” autism can be found in ASD. the authors observed a statistically significant decrease in the intrinsic frequency of sleep spindles in different brain regions in the AS group in relation to the NT development group, which may reflect the immaturity in brain regions related to the integration of sleep spindles.

Recently, Gorgoni^28^ et al. reviewed 18 articles published from 2006 to 2019 in respect of their findings on EEG microstructural sleep patterns and their possible relationship with cognitive functioning in children and adolescents with neurodevelopmental disorders. The authors did not notice a specific microstructural sleep EEG pattern in AS, or any relationship between the pattern they found and diurnal functioning. Some studies have shown a decrease in spindle density in different regions of the ASD brain individuals, according to the age group analysed. ASD adults have exhibited that variation only in the central region^29,30^, whereas in ASD children, the problem is specifically localized in the right prefrontal area^6,31^. The change in spindle activity observed in the study by Cebreros-Paniagua et al. could be a consequence of altered thalamo-cortical processes involved in spindle production in ASDs. The regional differences between children and adults could be associated with the atypical brain development that characterizes autism^32^. The observed central and prefrontal K-complex decrease in children with AS^6^ may represent an index of perturbation of the sleep protective mechanisms that involve K-complexes^33^. Alternatively, reduced K-complex density in the central and prefrontal regions may indicate possible neurodegenerative processes^34,35^. Some other findings^36-39^ suggest that changes in slow wave activity in ASD mirror the atypical cortical maturation detected in this population^32^, which was associated with thalamo-cortical alterations^37^ and a specific pattern in distinct age ranges.

To conclude, studies into the relationship between sleep problems and ASD are still at an early stage. Further research focusing on understanding and modifying the factors which contribute to sleep problems in ASD could make a significant contribution to improving the quality of life of this population. The studies described here published in this issue of Sleep Science represent a relevant contribution to help in this effort.
